# Investigation the determinants of pharmaceutical expenditure share of GDP in Iran and selected OECD countries

**DOI:** 10.1186/s40545-021-00371-2

**Published:** 2021-10-12

**Authors:** Azin Kadkhodamanesh, Vida Varahrami, Leila Zarei, Farzad Peiravian, Mohammad Hadidi, Nazila Yousefi

**Affiliations:** 1 School of Pharmacy, Shahid Beheshti University of Medical Sciences, Tehran, Iran; 2 Department of Economics, Shahid Beheshti University, Tehran, Iran; 3 Health Policy Research Center, Institute of Health, Shiraz University of Medical Sciences, Shiraz, Iran; 4 Department of Pharmacoeconomics and Pharma Management, School of Pharmacy, Shahid Beheshti University of Medical Sciences, Tehran, Iran; 5 School of Pharmacy, Shiraz University of Medical Sciences, Shiraz, Iran

**Keywords:** Pharmaceutical expenditure, Panel data, Time-series regression, Health system, Pharmaceutical system

## Abstract

**Aim:**

This study estimated the GDP share of pharmaceuticals in Iran based on the drivers of pharmaceutical expenditure and compared it with that of 31 members of the Organisation for Economic Cooperation and Development (OECD).

**Subject and methods:**

The factors contributing to pharmaceutical expenditure were identified through literature review and studied by 8 experts to classify the factors. Then, using the panel data method, a model was built to estimate the GDP share of pharmaceutical expenditure based on the extracted factors of the selected countries in Iran’s model. To explain the observed differences, several regression analyses were performed based on cross-sectional data. The analyses were performed using EVIEWS software, version 10.

**Results:**

The explanatory variables for the selected countries in the panel model (*R*^2^ = 0.98) were specified. Government health expenditure (β = 0.1432), the share of generic drugs (β = − 0.0143), gross domestic product (GDP) per capita (β = − 0.0058) and the rate of disability-adjusted life-years (DALY) (β = 0.0028) contributed most to pharmaceutical expenditure. In comparison, in the Iranian estimation model (*R*^2^ = 0.84), government health expenditure (β = 0.0536) and the share of generic drugs (β = 0.0369) had a significant impact on pharmaceutical expenditure. In the estimation model with more estimators for Iran (*R*^2^ = 0.99), government health expenditure (β = 0.1694), disease prevalence (β = 0.0537), the share of generic drugs (β = 0.0102), the DALY rate (β = 0.0039), GDP per capita (β = − 0.0033), and the drug price index (β = 0.0007) contribute most to pharmaceutical expenditure.

**Conclusion:**

In the models of the study, factors related to the structure of the healthcare system and the pharmaceutical system contributed most to pharmaceutical expenditure as a share of GDP. Moreover, disease profiles show its predictive role in the second model for Iran.

## Background

In recent decades, the cost of health care in general and pharmaceutical care in particular has been increasing due to the tremendous growth rate in the utilization of health care services, technological advancement, population growth, lifestyle changes resulting from industrialization, and the emergence of new diseases [14, 15]. This is an inevitable and logical process, with previous studies showing that advances in pharmaceutical technology lead to improvements in patients’ quality of life [9, 12].

Nevertheless, the sharp increase in drug costs has become one of the most important problems in health care, even in developed and rich countries [17]. Therefore, the financing of pharmaceutical expenditure has moved to the forefront of health policy and is also the greatest challenge [4, 16]. Most countries around the world are adopting a variety of strategies to control the growing healthcare expenditure, especially the part attributable to the cost of drugs, as controlling this part plays a crucial role in managing the overall healthcare expenditure. A review study conducted in 2014 showed that the most important factor that governments consider in controlling drug expenditure is factors related to price, utilization, therapeutic choice, demand and health care system [16].

Pharmaceutical expenditure as a percentage of GDP shows how much of the country’s total income is spent on the pharmaceutical sector [7]. Although pharmaceutical expenditure in the most OECD countries covers spending on prescription and over-the-counter medicines consumed as outpatient treatment [8], pharmaceutical expenditure in Iran is included pharmaceutical spending on outpatient and inpatient medicines. Empirical studies have shown that there is a strong relationship between GDP and pharmaceutical sector profits and health expenditure in general [19]. On the other hand, health expenditure and GDP have a reciprocal relationship in each country. Shaikh and Gandjour [21] pointed out that GDP has a strong positive impact on pharmaceutical expenditure, with an elasticity of more than one in countries with low pharmaceutical expenditure and countries with high economic freedom. On the other hand, public expenditure on medicines has a negative impact on GDP per capita, especially in countries with limited economic freedom [21].

In the OECD (Organisation for Economic Cooperation and Development) countries, health expenditure as a percentage of GDP has increased, but the extent of this phenomenon varies between these countries [6, 18]. In many of these countries, this growth is outpacing economic growth and it is forcing governments to spend a larger share of the budget on it. For example, in 2017, the largest GDP share of pharmaceutical expenditure is attributed to Greece, which is 2.2 percent, and the smallest share is attributed to Luxembourg and Denmark, whose share is 0.06 percent [11, 23]. Between 2007 and 2018, the largest growth in annual pharmaceutical expenditure belonged to South Korea, with Greece showing a negative trend in this area [11, 23].

**Fig. 1:**
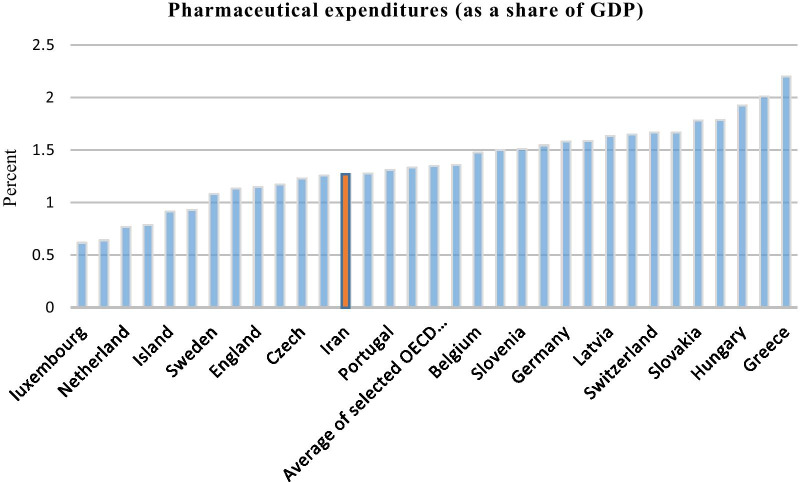
Comparison between pharmaceutical expenditure of Iran and the selected OECD countries (based on the [8])

Although, as shown in Fig. 1, the GDP share of pharmaceutical expenditure in Iran (calculated by dividing pharmaceutical expenditure (obtained from Iranian Pharmaceutical statistics datasheet^1^) into Iran GDP (obtained from World Bank database [25]) was lower than the average of this indicator in most OECD countries [3], it has shown an upward trend recently.

On the other hand, in recent years, especially after the Health Transition Plan (HTP), the government’s share in financing health and pharmaceutical expenditure has increased [24]. In some cases, this has resulted in the government and public insurance companies becoming indebted to suppliers, manufacturers, distributors and retailers of medicines, and the pharmaceutical supply chain being disrupted. Evidence shows that policy measures to control drug costs have a negative impact on the research and development process of the pharmaceutical industry. Therefore, in order to take effective policy measures, it is necessary to estimate pharmaceutical expenditures and evaluate the main factors contributing to pharmaceutical expenditure because policy makers should know the factors affecting costs in order to manage the budget [16].

The debate on pharmaceutical expenditure has generated a wide range of literature [4, 9, 22]. Nevertheless, from an aggregate perspective, there are very few studies that address the role of this expenditure in the GDP share of a country, and this work contributes to this debate. The objectives of this study are to estimate the GDP share of pharmaceuticals based on the drivers of pharmaceutical expenditure. Moreover, we intend to estimate this share for pharmaceutical expenditure in Iran.

## Methodology

### Study design

This retrospective study was conducted in 2019 and involved Iran and 31 OECD countries in the analysis. To achieve the research objectives, this study was conducted in four phases. In the first phase, the main factors contributing to pharmaceutical expenditure were identified and in the other phases, OECD and Iran models were estimated for the GDP share of pharmaceutical expenditure. To be more specific, in the second phase, a panel data method was used to develop a model to estimate the GDP share of pharmaceutical expenditure in the selected OECD countries. Then, in the third phase, based on the time-series regression analysis method and the selected variables, a model was developed to estimate the GDP share of pharmaceutical expenditure in Iran vis-à-vis that of the other countries in our sample. Then, in addition to our previous two models, another model with more estimators was presented to estimate the GDP share of pharmaceutical expenditure in Iran.

### Data collection

Due to the interdisciplinary nature of this study, related keywords were searched in Google scholar and Scopus databases. The search keywords were “Pharmaceutical Expenditure” OR “Health Expenditure” AND “Factor” OR “Variable”. Snowball method was used in order to complete search results. The search result yielded a total of 126 studies. After removing 25 duplicates and non-English, the title and abstract of the remaining 101 studies were screened for relevancy. Sixty-one obviously irrelevant studies were excluded. Forty citations were selected for full-text review, of which 21 studies were included. Then, model variables selected based on the literature and experts’ opinions considering the availability of data.

The factors contributing to pharmaceutical expenditure were identified through selected literatures and were examined by 8 health economists, third-party payers and pharmaceutical policy makers to review and classify the factors. The effective factors that emerged from the literature review were then refined and selected through expert interview. Table 1 shows the independent variables in the study models and the data sources.

**Table 1:** The independent variables in the study’s models

Category	Variable	Definition/scale	Source of data	In which model was used
Macroeconomic factors	GDP per capita	1/100 purchasing power parity (PPP) and fixed dollars (2011)	World Bank	The panel data model of OECD countries and the first and second time-series model of Iran
Health system structure	Share of government health expenditures	The percent of government health expenditures in total government expenditures	WHO	The panel data model of OECD countries and the first time-series model of Iran
		The percent of government health expenditures in GDP	World Bank	The second time-series model of Iran
Disease profiles	DALY disability-adjusted life-years	Per year for 100,000 population/this variable reflects the health status of the population	World Burden of Disease website	The panel data model of OECD countries and the first and second time-series model of Iran
	Disease prevalence	Number of patients in the total population	World Burden of Disease website	The second time-series model of Iran
Pharmaceutical system structure	Generics share	generics volume share of total pharmaceutical market volume	The OECD website, Pharmaceutical statistics yearbook of Iran	The panel data model of OECD countries and the first and second time-series model of Iran
	Pharmaceutical price index	Divide total pharmaceutical cost and total volume of numerical consumption annually	Pharmaceutical statistics yearbook of Iran	The second time-series model of Iran
Innovation	New drugs to market annually	Number of drug entered in the drug list each year	Iran drug list	The second time-series model of Iran

The OECD countries whose data were used for the study included Australia, Austria, Belgium, Canada, Czech Republic, Denmark, Finland, France, Germany, Greece, England, Hungary, Iceland, Ireland, Italy, Japan, South Korea, Luxembourg, Mexico, the Netherlands, Norway, Lithuania, Poland, Portugal, Slovakia, Spain, Sweden, Switzerland, Estonia, Slovenia and Latvia.

The data for the panel data models were obtained from 2008 to 2017. In addition, the time-series data for Iran were extracted for a period of 20 years (1998–2017), as we had more historical data available in the case of Iran.

### Data analysis

The variables extracted in the first phase were screened and classified into five general categories:

(1) Macroeconomic factors; (2) health system structure; (3) pharmaceutical system structure; (4) disease profiles; (5) innovation.

Then, the GDP share of pharmaceutical expenditure was estimated based on the above regressors in the three different models using EVIEWS software, version 10.

### The introduction of the models and their variables

Three models were constructed to estimate the factors influencing the OECD and Iran on their GDP share of pharmaceutical expenditure.

#### Estimation of the GDP share of the pharmaceutical expenditure of the OECD countries using the panel data method

The basic panel data framework is as follows:

$$Y\_{\text{it}} = \, \alpha \_{\text{i}} + \, \beta {\text{X}}\_{\text{it}} + {\text{ U}}\_{\text{it}},$$

where α is the constant, β is the coefficient, and Uit is the error term U_it ~ N(0.δ^2). In this model, Yit represents a panel data observation. Yit is observed for all individuals i = 1, …, N over all time periods *t* = 1, … ,T.

In the panel data model of the OECD countries, the share of pharmaceutical expenditure was estimated for the selected OECD countries based on data access. In this model, GDP per capita was used as a proxy for macroeconomic factors. Since it is considered in terms of PPP and the dollar value in 2011, the effect of price changes and inflation has not been not taken into account (the so-called real GDP).

The data for each variable were entered into the software for conducting panel data analysis tests, and required analyses, namely reliability test, aggregate test, F-Limer test and Hausman test, were conducted.

#### Estimation of the GDP share of pharmaceutical expenditure in Iran using the time-series method

In the comparative estimation model for Iran, the variables mentioned in the previous model were used as independent variables to compare the two models. GDP share of pharmaceutical expenditure as dependent variable, were calculated by dividing Iran’s pharmaceutical expenditures (obtained from Iranian pharmaceutical statistical datasheet published by the Iranian Ministry of Health^2^) into Iran’s GDP (obtained from World Bank database [25]). To perform the time-series regression analysis in this study, the Segmented Regression Interrupted Time-Series model was used.

To test the time-series regression assumptions and model validity, required analyses namely, reliability, residual normality, autocorrelation, and heterogeneity tests were conducted.

Then, given the limitations in the selection of variables for the comparative model for Iran, in another model, the GDP share of pharmaceutical expenditure in Iran was examined separately with an extended set of independent variables.

#### Estimating the GDP share of pharmaceutical expenditure in Iran with more variables

In this model, in addition to the variables mentioned in the previous models, pharmaceutical price index, disease prevalence, and new drugs entering the market each year were considered as independent variables.

The data for each variable for Iran were entered into the software for the statistical analysis of time-series regression analysis. Other required analyses were performed, namely, reliability study, residual normality study, autocorrelation study, and heterogeneity analysis. The results are presented in the next section.

## Findings

### Estimation of the GDP share of pharmaceutical expenditure in the OECD countries using the panel data method

The Levin–Lin-Chu (LLC) test was used for the existence of a unit root. The results showed that all variables were stationary in level.

The F-Limer test was used to test the validity of the panel data model against the pooled model. The results of the F-Limer test showed that the *p*-value is less than the significance level of 0.05 (probability = 0.0001); therefore, the panel data model was used to estimate the proportion of the pharmaceutical expenditure of the OECD countries. In addition, the Hausman test was used to choose between the fixed-effect approach and the random-effect approach. The results confirmed the fixed-effect model (probability = 0.0001). Thus, pharmaceutical expenditure (as a share of GDP) in the OECD countries was examined using the fixed-effect panel data approach. The results are presented in Table 2.

**Table 2:** Results from the estimation model of OECD countries from 2008 to 2017 Source: EVIEWS software, version 10

Independent variables	Coefficient	*t*-Statistic	Probability
C	1.1942	1.211	0.2308
GDP	− 0.0058	− 5.5823	0.0000
GHE	0.1432	7.1777	0.0000
DALY	0.0028	2.0726	0.0428
GENERICVOL	− 0.0143	− 4.8635	0.0000
*R*-squared: 0.9794		Probability (*F*-statistic): 0.0001	

The value of *R*-squared (*R*^2^) confirms that the model was a good fit. The *R*^2^ value indicates that the model was able to explain 97.94% of the variance present in the data. Thus, based on the data collected, an estimation model for the share of pharmaceutical expenditure in GDP of OECD countries could be:

$$Y = {1}.{1942 } - \, 0.00{58}*{\text{GDP }} + \, 0.{1432}*{\text{GHE }} + \, 0.00{28}*{\text{DALY }} - \, 0.0{143}*{\text{GENERICVOL}},$$

where *Y* = the share of pharmaceutical expenditure in GDP for OECD countries; GDP = gross domestic product per capita; GHE = government health expenditure (% of total expenditure); DALY = the rate of disability-adjusted life-years, and GENERICVOL = generic drug volume as a percentage of total pharmaceutical market volume.

The following is an analysis of the above equation results:

1. GDP has a positive impact on pharmaceutical spending (as a share of GDP). The GDP regression coefficient of the test shows that when GDP increases by 1 unit, Y decreases by 0.0058 units.
2. The GHE regression coefficient is + 0.1432, which means that this variable has a positive effect on the dependent variable, and if GHE increases by 1 unit, then the dependent variable will increase by 0.1432 units.
3. DALY has a positive correlation with the dependent variable. The DALY regression coefficient of the test is + 0.0028, which means that if DALY increases by 1 unit, then the dependent variable increases by 0.0028 units.
4. GENERICVOL has a negative effect on the dependent variable. The results show that when GENERICVOL increases by 1 unit, the dependent variable decreases by − 0.0143 units.

### Estimation of the GDP share of pharmaceutical expenditure in Iran using the time-series method

To evaluate the presence of the unit root, augmented Dicky–Fuller (ADF) test was used. The results showed that *Y*, GDP, GHE and DALY are stationary in first differences; however, GENERICVOL is stationary in level. Since one variable was stationary in level, while others were stationary in the first difference, the co-integration test was necessary. The results of the co-integration test showed that the variables were co-integrated.

Therefore, compared to the previous model, pharmaceutical expenditure (as a share of GDP) in Iran was evaluated over the period 1999–2017 using a time-series regression model, and the following results were obtained (Table 3).

**Table 3:** Results from the estimation model of Iran from 1999 to 2017 Source: EVIEWS software, version 10

Independent variables	Coefficient	*t*-Statistic	Probability
C	− 4.1597	− 2.2993	0.0443
GDP	0.0001	0.1119	0.9131
GHE	0.0536	5.5145	0.0003
DALY	0.0019	0.6789	0.5126
GENERICVOL	0.0369	2.7862	0.0192
*R*-squared: 0.8442	Probability (*F*-statistic): 0.0001		D.W stat: 2.3749

The residuals of the equation were tested for the presence of non-normality, heteroscedasticity, and serial correlation. The results showed that the model does not have the problem of non-normality, heteroscedasticity, and autocorrelation. The value of *R*-squared (*R*^2^) confirmed that the model was a good fit. The *R*^2^ value indicated that the model was able to explain 84.42% of the variance present in the data. Thus, based on the results, an estimation model for the GDP share of pharmaceutical expenditure in Iran could be:

$$Y = - {4}.{1597} + 0.000{1}*{\text{GDP }} + \, 0.0{536}*{\text{GHE}} + \, 0.00{19}*{\text{DALY }} + \, 0.0{369}*{\text{GENERICVOL}}.$$

The following is an analysis of the equation:

1. In this estimation model with this set of factors, GDP has no significant effect on the dependent variable (*p*-value > 0.05).
2. The positive coefficient of the GHE shows that this variable has a positive effect on Iran’s pharmaceutical expenditure (as a share of GDP). A one-unit increase in the GHE could lead to a 0.0536 increase in the dependent variable.
3. DALY has no significant effect on the dependent variable in this model (*p*-value > 0.05).
4. The positive coefficient of GENERICVOL shows that this variable has a positive and significant effect on Iran’s pharmaceutical expenditure (as a share of its GDP). This means that a one-unit increase in GENERICVOL could lead to a 0.0369 increase in the dependent variable.

### Estimating the GDP share of pharmaceutical expenditure in Iran using other variables

For the quantitative assessment of pharmaceutical expenditure (as a share of GDP) in this estimation model, the dependent variable was determined by another set of independent variables.

The results of the unit root test (ADF) showed that Y, GDP, GHE, GENERICVAL, PRICE, INNOVATION and DALY are stationary in first differences. However, PREVALENCE is stationary in level. Since one variable is stationary in level while others are stationary in the first difference, the co-integration test was necessary. The results of the co-integration test showed that the data are co-integrated across variables.

Therefore, pharmaceutical expenditure (as a share of GDP) in Iran over the period 1999–2017 was evaluated using a time-series regression model. The results are presented below (Table 4).

**Table 4:** Results from the second estimation model of Iran from 1999 to 2017 Source: EVIEWS software, version 10

Independent variables	Coefficient	*t*-Statistic	Probability
C	− 52.4201	− 4.9740	0.0006
GDP	− 0.0033	− 3.5449	0.0053
GHE	0.1694	8.5718	0.0000
DALY	0.0039	4.0904	0.0022
GENERICVOL	0.0102	3.6207	0.0047
PRICE	0.0007	4.1996	0.0018
INNOVATION	0.0002	1.0365	0.3244
PREVALENCE	0.0537	4.9530	0.0006
*R*-squared: 0.9967	Probability (*F*-statistic): 0.0001		D.W stat: 2.4733

The tests related to non-normality, autocorrelation and heteroscedasticity of the equation residuals showed that the model did not have the problem of non-normality, serial correlation, and heteroscedasticity. The model was able to explain up to 99% of the variance present in the data (*R*^2^ = 0.99); therefore, it was accepted as a good fit. The estimation model of Iran's pharmaceutical expenditure yielded the following equation:

$$Y = - {52}.{42} - 0.00{33}*{\text{GDP}} + 0.{1694}*{\text{GHE}} + 0.0{1}0{2}*{\text{GENERICVAL}} + 0.000{7}*{\text{PRICE}} + 0.000{2}*{\text{ INNOVATION }} + \, 0.00{39}*{\text{DALY}} + \, 0.0{537}*{\text{PREVALENCE}},$$

where *Y* = Iran’s pharmaceutical expenditure (as a share of its GDP); GDP = gross domestic product per capita; GHE = government health expenditure (as a % of GDP); GENERICVAL = the value of generic drugs as a share of the total value of the pharmaceutical market; PRICE = price index; INNOVATION = new drugs entering the market each year; DALY = the rate of disability-adjusted life-years, and PREVALENCE = prevalence of disease.

The following is an analysis of the equation:

1. GDP has a negative effect on the dependent variable. The GDP regression coefficient of the test is − 0.0033, which means that a one-unit increase in GDP leads to a 0.0033 decrease in the dependent variable.
2. The GHE has a positive correlation with the dependent variable. The regression coefficient of the GHE is 0.1694, which means that a one-unit increase in GHE leads to a 0.1694 increase in the dependent variable.
3. GENERICVAL has a positive effect on the dependent variable. The regression coefficient of this variable indicates that when GENERICVAL increases by 1 unit, the dependent variable increases by 0.0102 units.
4. The PRICE has a positive effect on the dependent variable. The regression coefficient of this variable indicates that when PRICE increases by 1 unit, the dependent variable increases by 0.0007 units.
5. INNOVATION has no significant effect on the dependent variable in this model (*p*-value 0.05).
6. DALY has a positive correlation with the dependent variable. The regression coefficient of DALY is 0.0039, which means that if DALY increases by 1 unit, then the dependent variable increases by 0.0039 units.
7. PREVALENCE has a positive effect on the dependent variable in this model. The regression coefficient of this variable indicates that if PREVALENCE increases by 1 unit, then the dependent variable will increase by 0.0537 units.

## Discussion

This study set out to estimate the relationship between pharmaceutical expenditure (as a share of GDP) and the factors affecting this proportion in Iran and the selected OECD countries. For this purpose, a panel data model was used for the OECD countries and a time-series regression model was used for Iran vis-à-vis the selected OECD countries. In addition, a second time-series regression model with an extended set of factors was used for Iran.

The results of the panel model for the OECD countries show that government health expenditure as a percentage of the total expenditure (β = 0.1432), the share of generic drugs in the pharmaceutical market (β = -0.0143), GDP per capita (β = − 0.0058), and the rate of disability-adjusted life-years (β = 0.0028) contribute most to pharmaceutical expenditure (as a share of GDP) (the conceptual model for the estimation model of the selected OECD countries is shown in Fig. 1). In comparison, in our first estimation model for Iran (R2 = 0.84), of these four variables, government health expenditure as a percentage of the total expenditure (β = 0.0536) and the share of generic drugs in the pharmaceutical market (β = 0.0369) had a significant effect on pharmaceutical expenditure (as a share of GDP). In both models, factors related to the structure of the health care system (the GHE variable) and the structure of the pharmaceutical system (the GENERICVAL variable) are important determinants of the share of pharmaceutical expenditures in the GDP. The government's share of health expenditure was used to explain the government’s financial participation in health expenditure. This variable had the largest regression coefficient in both estimation models. This result is consistent with the findings of Hsieh et al. [10] and Roy et al. [20], who suggest that an increase in government financial participation in health spending can improve access to health services, which in turn can increase health and pharmaceutical spending. Majority of health expenditures in Iran including pharmaceutical expenditure are reimbursed by Social Security Organization (public), Medical Services Insurance Organization (governmental), Medical Insurance Services Organization of Armed Forces (governmental), and supplementary private insurances [5].

In addition, the share of generic drugs was used as a quantitative indicator of the drug market structure and prescribing practices. In our estimation model for the selected OECD countries, this variable has a negative impact on the dependent variable. This result is consistent with the findings of Andersson et al. [1] and Lu et al. [13] and supports the fact that switching the drug market to generics can go a long way toward controlling pharmaceutical expenditures.

**Fig. 2:**
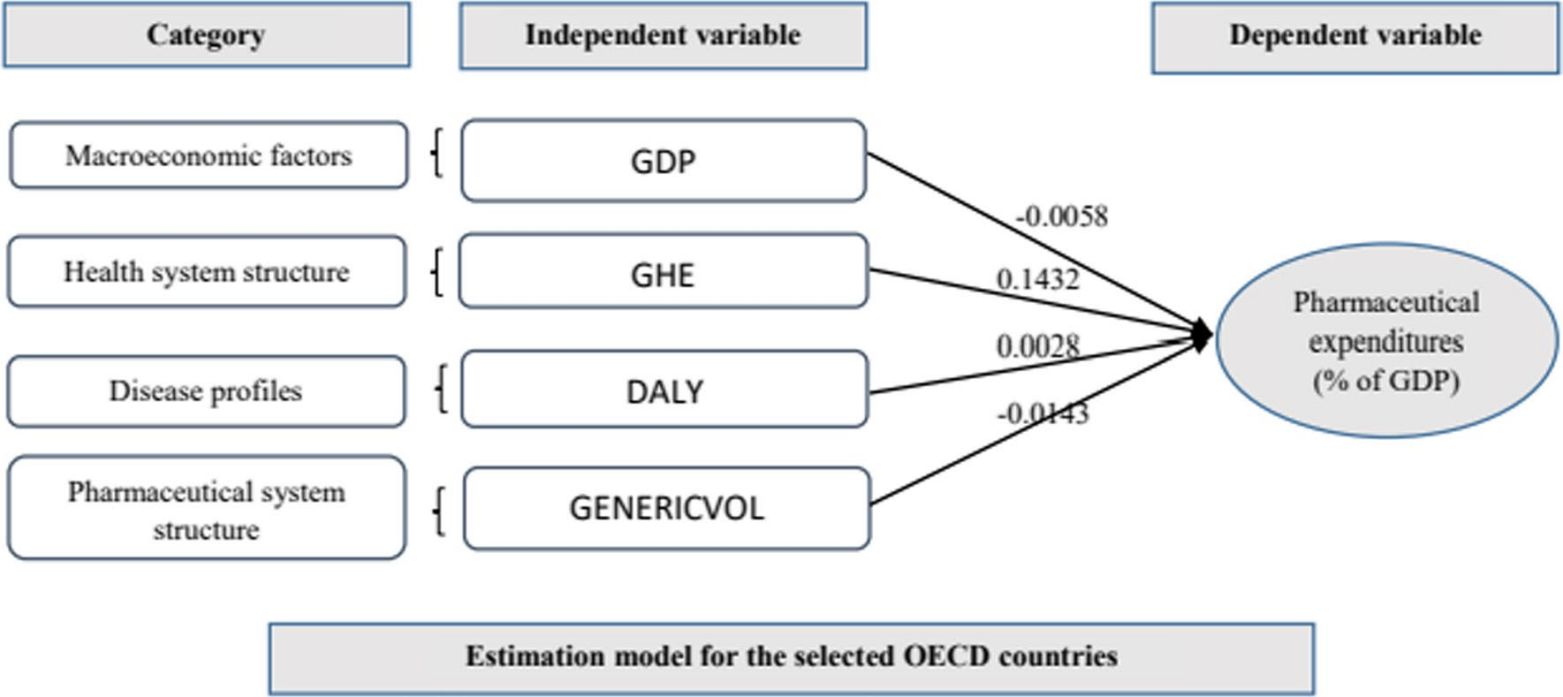
Conceptual model for the pharmaceutical expenditure of the selected OECD countries

Due to some limitations in data collection in the estimation models for Iran, locally manufactured drugs were considered as generic drugs. The results show that the share of generic drugs positively affects pharmaceutical expenditures (as a share of GDP). This positive relationship can be partly explained by the response of domestic pharmaceutical companies to price control policies; these companies introduced a new branded generic drug into the pharmaceutical market and, subsequently, encouraged prescribers to prescribe more expensive drugs. Another important reason lies in Iran’s main policy. Pharmaceutical policy in Iran is based on generic drug policy and has some differences compared to the policy of the OECD countries. In these countries, branded drugs command a very large share in drug expenditure and switching to generic drugs logically leads to cost reduction. But in Iran, higher use of generics also means higher costs because of the large share of generic drugs in drug expenditure (Fig. 2).

The results of the second estimation model for Iran with more variables show that government health expenditure as a share of GDP (β = 0.1694), disease prevalence (β = 0.0537), generic drugs’ share of the pharmaceutical market (β = 0.0102), rate of disability-adjusted life-years (β = 0.0039), GDP per capita (β = − 0.0033), and pharmaceutical price index (β = 0.0007) contribute most to pharmaceutical expenditure (as a share of GDP). The innovation variable had no significant effect in this model, where factors related to health system structure, disease profiles and pharmaceutical system structure contributed most to pharmaceutical expenditure as a share of GDP in Iran.

Factors related to disease profile (DALY and PREVALENCE) had a positive correlation with pharmaceutical expenditure (as a share of GDP). This result is in good agreement with the results of Roy et al. [20]. However, the result of the innovation variable is, however, at variance with that of a study by Awad et al. [2] who indicated that innovation is an important factor affecting drug expenditure. Most innovations in Iran have been carried out in recent years; therefore, it is necessary to evaluate the effect of innovation in the long run.

**Fig. 3:**
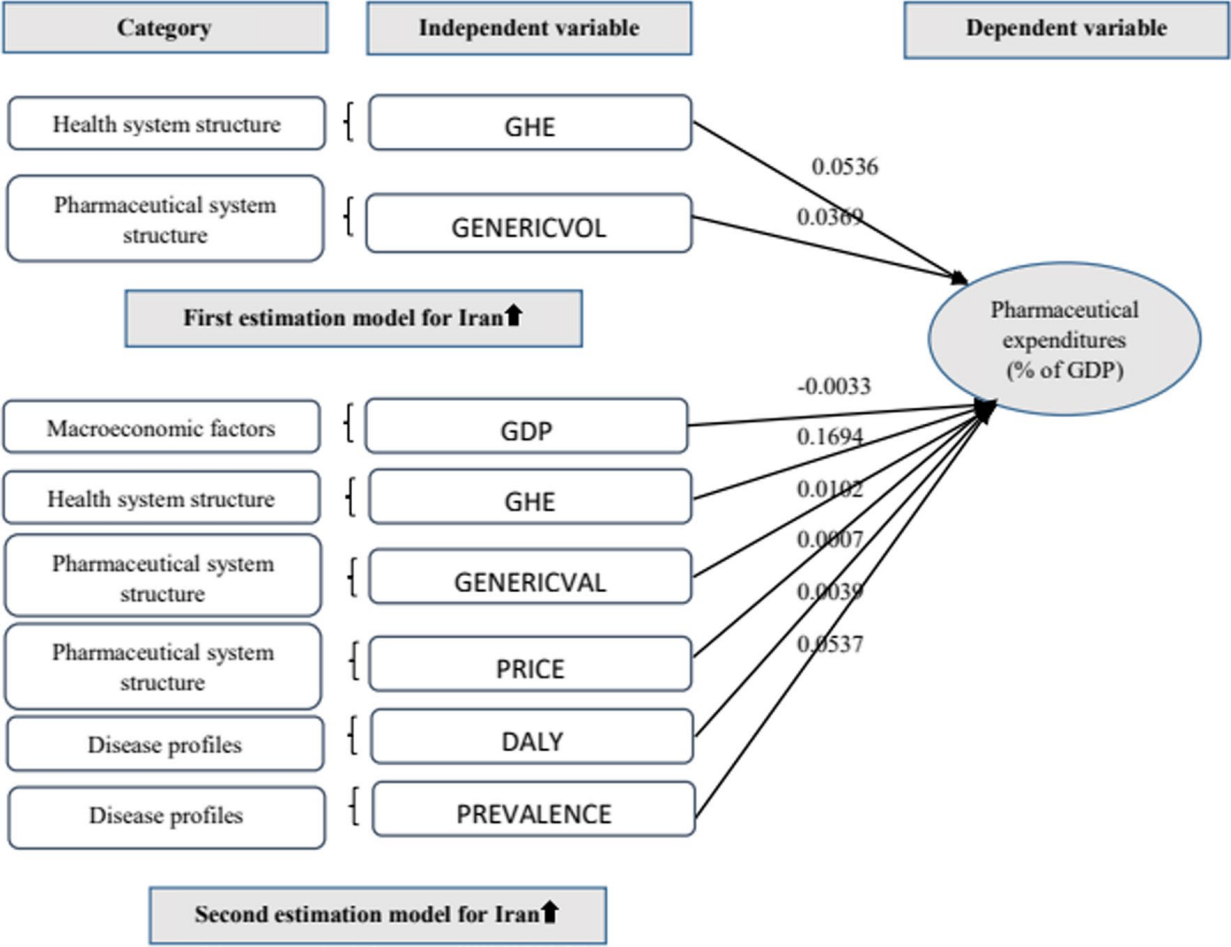
Conceptual model for Iran's pharmaceutical expenditure

The conceptual model used to estimate the models for Iran is constructed as follows (Fig. 3).

## Conclusion

In our model of the selected OECD countries and Iran, factors related to the structure of the health care system and the structure of the pharmaceutical system contributed most to the GDP share of pharmaceutical expenditure. Moreover, in the second estimation model of Iran, the disease profiles of the country were added as a contributing factor to the GDP share of pharmaceutical expenditure in this country.

## Data Availability

All raw data are available in the formal sources. In addition, the datasets used and/or analyzed during the current study available from the corresponding author on reasonable request.

## Footnotes

1 - https://www.fda.gov.ir/fa/%D8%A7%D8%B7%D9%84%D8%A7%D8%B9%DB%8C%D9%87%E2%80%8C%D9%87%D8%A7/%D8%A7%D9%85%D8%A7%D8%B1%D9%86%D8%A7%D9%85%D9%87-%D8%AF%D8%A7%D8%B1%D9%88%DB%8C%DB%8C-%D8%B3%D8%A7%D9%84-1399

2 - https://www.fda.gov.ir/fa/%D8%A7%D8%B7%D9%84%D8%A7%D8%B9%DB%8C%D9%87%E2%80%8C%D9%87%D8%A7/%D8%A7%D9%85%D8%A7%D8%B1%D9%86%D8%A7%D9%85%D9%87-%D8%AF%D8%A7%D8%B1%D9%88%DB%8C%DB%8C-%D8%B3%D8%A7%D9%84-1399
